# Methylomic survival predictors, frailty, and mortality

**DOI:** 10.18632/aging.101392

**Published:** 2018-03-06

**Authors:** Yan Zhang, Kai-Uwe Saum, Ben Schöttker, Bernd Holleczek, Hermann Brenner

**Affiliations:** 1 Division of Clinical Epidemiology and Aging Research, German Cancer Research Center (DKFZ), Heidelberg D-69120, Germany; 2 German Cancer Consortium (DKTK), German Cancer Research Center (DKFZ), Heidelberg D-69120, Germany; 3 Network Ageing Research, University of Heidelberg, Heidelberg 69115, Germany; 4 Saarland Cancer Registry, Saarbrücken D-66119, Germany; 5 Division of Preventive Oncology, German Cancer Research Center (DKFZ) and National Center for Tumor Diseases (NCT), Heidelberg D-69120, Germany

**Keywords:** mortality risk score, epigenetic clock, frailty, mortality

## Abstract

Survival predictors are of potential use for informing on biological age and targeting prevention of aging-related morbidity. We assessed associations of 2 novel methylomic survival indicators, a methylation-based mortality risk score (MRscore) and the epigenetic clock-derived age acceleration (AA), with a well-known survival predictor, frailty index (FI), and compared the 3 indicators in mortality prediction. In a large population-based cohort with 14-year follow-up, we found both MRscore and AA to be independently associated with FI, but the association was much stronger for MRscore than for AA. Although all 3 indicators were individually associated with all-cause mortality, robust associations only persisted for MRscore and FI when simultaneously including the 3 indicators in regression models, with hazard ratios (95% CI) of 1.91 (1.63–2.22), 1.37 (1.25–1.51), and 1.05 (0.90–1.22), respectively, per standard deviation increase of MRscore, FI, and AA. Prediction error curves, Harrell’s C-statistics, and time-dependent AUCs all showed higher predictive accuracy for MRscore than for FI and AA. These findings were validated in independent samples. Our study demonstrates the ability of the MRscore to strongly enhance survival prediction beyond established markers of biological age, such as FI and AA, and it thus bears potential of a surrogate endpoint for clinical research and intervention.

## Introduction

With the population aging worldwide [1], preservation of good health at older ages has become one of the most important public health challenges and development of interventions that can counteract aging-related morbidity and mortality is emerging as major area of research. This necessitates a good measure of individual’s biological age to assess the benefits from interventions. Frailty indices (FI), based on the accumulation of declines in health and function ability, which are typically expressed as proportion of age-related health deficits presented from a list of such deficits, are regarded as one of best characterized measures of biological age [2, 3]. They are closely related to chronological age and other aging-related phenotypes [4-6], and predict longevity better than chronological age [7]. Another attractive indicator of biological age is the recently established epigenetic clock, also known as DNA methylation (DNAm) age, which was trained to be highly correlated to chronological age but estimates the biological age of a tissue, cell or organ based on DNAm of multiple CpGs across the genome [8]. The deviation of thus derived DNAm age, i.e. the epigenetic clock, from the chronological age is termed epigenetic age acceleration (AA). The AA was found to be predictive for mortality, independent from chronological age [9-11]. A growing body of evidence also indicates associations between the AA and various aging-related diseases [12-15], as well as FI [16]. However, a recent study comparing the FI and AA side by side for survival prediction demonstrated that the FI outperformed the AA, and the AA was not a significant predictor in the presence of FI [17]

Recently, using an epigenome-wide approach, we derived and validated another robust predictor for survival, i.e. a mortality risk score (MRscore) based on 10 blood DNAm markers [18]. Our multivariate analyses showed that the strong association of the MRscore with mortality was independent from not only chronological age but also the epigenetic AA. Also, the significant association of the AA with mortality disappeared when adjusting for the MRscore. To further verify if the MRscore can serve as a reliable measure of biological aging, we simultaneously assessed the two methylomic survival predictors, MRscore and AA, in relation to a FI, as well as the individual and joint predictive values of the three indicators, i.e. MRscore, AA, and FI, for all-cause mortality in three subsets of a large population-based cohort of older adults with 14 years of follow-up.

## RESULTS

Altogether 993, 858, and 470 subjects with available data on MRscore, DNAm age and frailty were included in the analyses of subset I, II, and III, respectively. Table 1 shows the participants’ characteristics and average levels of the 3 survival indicators in the three subsets. Due to over-sampling of deceased participants in subset II and of participants with cancer diagnosis during follow-up in subset III, mean age, DNAm age, AA, and MRscore were higher in subset II and III than in subset I, while essentially no difference in FI was observed between the three subsets. The proportions of current smokers were also higher in subset II and III than in subset I. During follow-up, 264 participants included in subset I died, subset II included 435 deaths, of which 120 were from the subcohort (N=543), and 199 participants in subset III died (Supplementary Figure S1).

**Table 1:** Description of study population.

Characteristics	Subset I (n=993)	Subset II (n=858)	Subset II (n=470)
Age (years; mean ± SD)	62.1 ± 6.5	62.9 ± 6.7	63.2 ± 6.2
Methylation age (years; mean ± SD)^a^	61.6 ± 7.1	64.6 ± 7.7	66.7 ± 6.8
Age acceleration (years; mean ± SD)^b^	-1.0 ± 4.9	1.2 ± 5.2	0 ± 4.8
Sex (N/%)			
Men	494 (49.8)	390 (45.4)	258 (54.9)
Women	499 (50.2)	468 (54.6)	212 (45.1)
Educational levels (N/%)			
Low (≤ 9 years)	736 (74.1)	657 (76.6)	358 (77.5)
Intermediate (10 – 11 years)	155 (15.6)	124 (14.4)	64 (13.8)
High (≥12 years)	102 (10.3)	77 (9.0)	40 (8.7)
Body mass index (N/%)			
Underweight (<18.5 kg/m^2^)	8 (0.8)	5 (0.6)	3 (0.7)
Normal weight (18.5-<25.0 kg/m^2^)	240 (24.2)	258 (30.1)	127 (27.0)
Overweight (25.0-<30.0 kg/m^2^)	481 (48.4)	364 (42.4)	221 (47.0)
Obesity (≥30.0 kg/m^2^)	264 (26.6)	231 (26.9)	119 (25.3)
Smoking status (N/%)			
Never smoker	482 (48.6)	392 (45.7)	174 (38.1)
Former smoker	328 (33.0)	294 (34.3)	165 (36.1)
Current smoker	183 (18.4)	172 (20.0)	118 (25.8)
			
MRscore (mean ± SD)^c^	2.49 ± 2.29	3.30 ± 2.53	4.42 ± 2.56
cont.MRscore (mean ± SD)^d^	-2.65 ± 0.48	-2.51 ± 0.50	-2.30 ± 0.49
Frailty index (mean ± SD)	25% ± 15%	25% ± 15%	26% ± 16%

Abbreviations: cont.MRscore, continuous mortality risk score; MRscore, mortality risk score; SD; standard deviation.

^a^DNA methylation age calculated using Horvarth’s algorithm. ^b^Age acceleration estimated by the residuals of DNA methylation age regressed on chronological age. ^c^MRscore based on aberrant methylation of 10 CpGs (cg01612140, cg05575921, cg06126421, cg08362785, cg10321156, cg14975410, cg19572487, cg23665802, cg24704287, cg25983901): 0-10 refer to simultaneously aberrant methylation at 0 to 10 CpGs. ^d^cont.MRscore refers to a risk score computed through linear combination of weighted methylation values of the 10 CpGs.

### Associations of MRscore/cont.MRscore and age acceleration with FI

Figure 1a, 1b, and 1c display the mutual correlations of age, DNAm age, epigenetic AA, MRscore, continuous MRscore (cont.MRscore), and FI in the three subsets, respectively. AA was moderately correlated with DNAm age (ρ=0.59, ρ=0.61, and ρ=0.63, respectively) but not with age (ρ=-0.05, ρ=0.0007 and ρ=-0.01, respectively), although age and DNAm age were highly correlated (ρ=0.75, ρ=0.76 and ρ=0.74, respectively). The two forms of mortality risk score, i.e. MRscore and cont.MRscore, were highly correlated (ρ=0.83, ρ=0.85 and ρ=0.86, respectively), and their correlations with AA (ρ=0.24 for MRscore and ρ=0.27 for cont.MRscore in subset I and II; ρ=0.20 for MRscore and ρ=0.19 for cont.MRscore in subset III) were higher than with FI (ρ=0.12-0.19). Correlations between AA and FI were very low (ρ=0.05, ρ=0.08 and ρ=0.09, respectively).

**Figure 1a:**
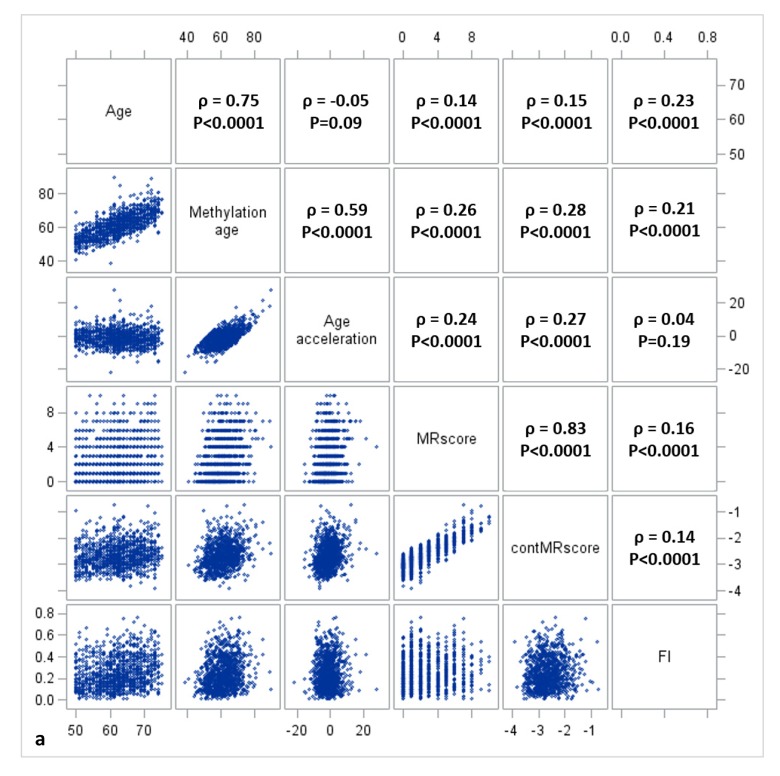
**Correlation matrix of methylomic survival predictors and frailty in subset I**.

**Figure 1b:**
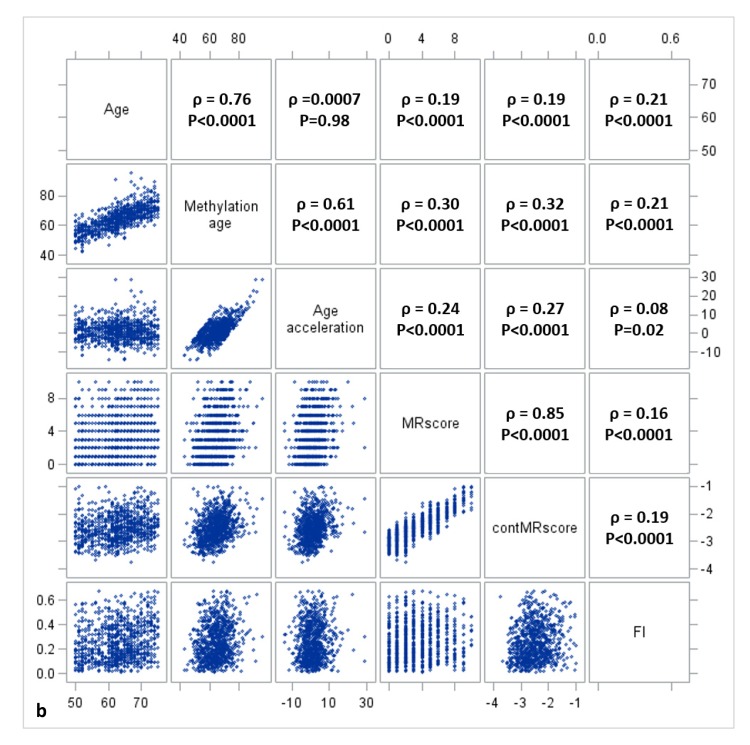
**Correlation matrix of methylomic survival predictors and frailty in subset II**.

**Figure 1c:**
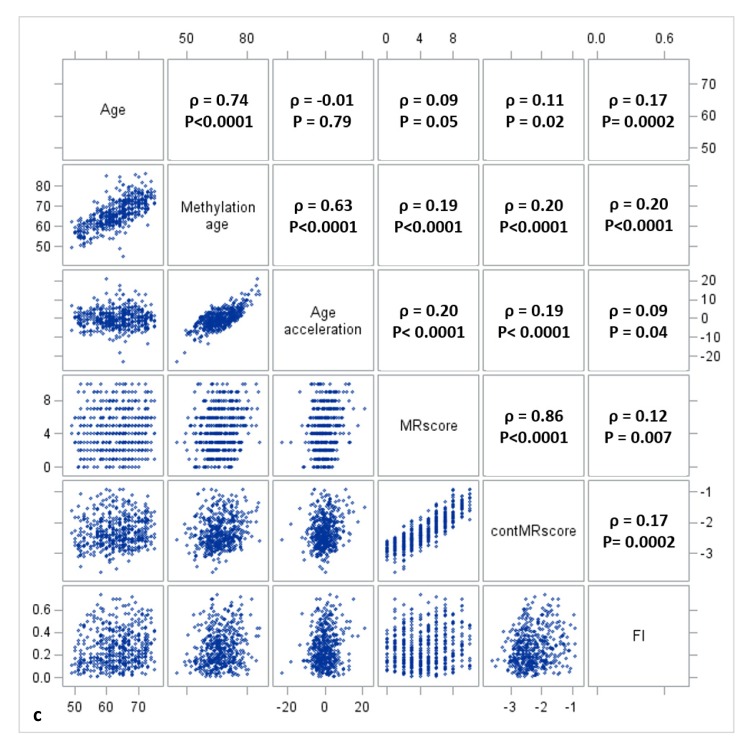
**Correlation matrix of methylomic survival predictors and frailty in subset III**.

Table 2 shows the associations of MRscore/ cont.MRscore and AA with FI estimated by regression models. Results from the three subsets were generally very consistent. According to the meta-analysis of overall samples, FI increased by 2.2% units per standard deviation (SD) increase in MRscore, by 3.1% units per SD increase in cont.MRscore, and by 1.4% units per 5-year (SD) AA (Table 2, Model 2). Simultaneously including mortality risk score (either MRscore or cont.MRscore) and AA into the regression model (model 3) attenuated the association estimates, but statistically significant associations persisted for all indicators in the meta-analysis of the three subsets.

**Table 2:** Associations of methylomic survival predictors with frailty index (coefficients are reported per increase in the predictor by one standard deviation).

Methylation marker	Model type^a^	Subset I (n=993)			Subset II (n=858)			Subset III (n=470)			Overall (meta-analysis)	
		Coefficient (95% CI)^b^	p-value		Coefficient (95% CI)^b^	p-value		Coefficient (95% CI)^b^	p-value		Coefficient (95% CI)^b^	p-value
MRscore^c^	Model 1	2.30 (1.22, 3.38)	2.94E-5		1.71 (0.54, 2.88)	4.20E-3		1.91 (0.45, 3.38)	0.01		2.01 (1.31, 2.70)	1.85E-8
	Model 2	2.81 (1.60, 4.01)	4.95E-6		1.95 (0.64, 3.27)	3.62E-3		1.42 (-0.29, 3.13)	0.10		2.21 (1.42, 3.00)	4.18E-5
												
cont.MRscore^d^	Model 1	2.70 (1.39, 4.01)	5.24E-5		2.53 (1.18, 3.89)	2.54E-4		2.68 (1.21, 4.15)	2.56E-4		2.64 (1.84, 3.43)	7.22E-11
	Model 2	3.55 (2.00, 5.10)	7.37E-6		3.40 (1.78, 5.04)	4.12E-5		2.09 (0.38, 3.80)	0.02		3.06 (2.12, 4.00)	1.62E-10
												
Age acceleration^e^	Model 1	1.30 (0.32, 2.28)	0.01		1.38 (0.34, 2.43)	0.01		1.86 (0.44, 3.29)	0.01		1.45 (0.80, 2.09)	9.26E-6
	Model 2	1.28 (0.30, 2.25)	0.01		1.38 (0.33, 2.42)	0.01		1.50 (0.02, 2.99)	0.04		1.36 (0.72, 2.00)	3.41E-5
												
MRscore^c^	Model 3	2.56 (1.31, 3.80)	5.66E-5		1.65 (0.30, 3.00)	0.02		1.21 (-0.51, 2.93)	0.17		1.93 (1.13, 2.74)	2.71E-6
Age acceleration^e^	Model 3	0.77 (-0.24, 1.78)	0.13		1.06 (-0.01, 2.13)	0.05		1.39 (-0.12, 2.91)	0.07		1.00 (0.33, 1.66)	3.03E-3
												
cont.MRscore^d^	Model 3	3.21 (1.62, 4.82)	7.98E-5		3.08 (1.41, 4.75)	3.06E-4		1.91 (0.19, 3.63)	0.04		2.77 (1.81, 3.72)	1.56E-8
Age acceleration^e^	Model 3	0.81 (-0.20, 1.81)	0.12		0.90 (-0.16, 1.97)	0.10		1.34 (-0.17, 2.85)	0.08		0.94 (0.29, 1.60)	4.93E-3

Abbreviations: CI, confidence interval; cont.MRscore, continuous mortality risk score; MRscore, mortality risk score.

^a^ Model 1: adjusted for age, sex, leukocyte composition; Model 2: similar as Model 1, additionally adjusted for smoking status and alcohol consumption; Model 3: similar as Model 2, additionally adjusted for epigenetic clock/mortality risk score. ^b^Estimated for changes (95% confidence) in frailty index expressed in % per unit of methylation markers. ^c^MRscore based on aberrant methylation of 10 CpGs (cg01612140, cg05575921, cg06126421, cg08362785, cg10321156, cg14975410, cg19572487, cg23665802, cg24704287, cg25983901): 0-10 refer to simultaneously aberrant methylation at 0 to 10 CpGs. ^d^cont.MRscore refers to a risk score computed through linear combination of weighted methylation values of the 10 CpGs. ^e^Age acceleration estimated by the residuals of DNA methylation age (estimated using Horvarth’s algorithm) regressed on chronological age.

### Associations between individual mortality-related CpGs and FI

The associations between individual mortality-related CpGs and FI are presented in Supplementary Table S2. Of 58 candidates identified in our previous study [18], 34 CpGs were also associated with frailty based on results from meta-analysis of the three subsets. The vast majority (n=31 CpGs) was inversely associated with FI, which increased by 1.5 to 9.6 % units per 10% units decrease in methylation. Only for 3 CpGs (i.e. cg23842572 in MPRIP, cg08362785 in MKL1, and cg04987734 in CDC42BPB), methylation was positively associated with FI, and FI increased by 3.2 to 4.9 % units per 10 % units decrease in methylation of the 3 CpGs. The CpG sites whose methylation was most strongly associated with FI were two CpGs located at SLC1A5 (cg01406381 and cg07626482), followed by cg19859270 in GPR15 and cg19266329 in 1q21.1.

### Associations of MRscore/cont.MRscore, age acceleration, and FI with all-cause mortality

Table 3 shows the individual and joint associations of MRscore/cont.MRscore, epigenetic AA, and FI with all-cause mortality. In meta-analysis of subset I and II (results for each individual subset are provided in Supplementary Table S3), HRs (95% CI) for participants with score of 1, 2-5, and 5+, respectively, were 1.65 (1.14 – 2.40), 2.23 (1.59 – 3.13), and 4.46 (2.96 – 6.73), compared to participants with score=0. Additional adjustment for AA and FI did not materially diminish the risk estimates. Likewise, risk of dying increased 4.4-fold per unit increase in cont.MRscore, 1.2-fold per 5-year AA, and 1.3-fold per 10 % units increase in FI. When HRs were expressed per increase of the predictors by one standard deviation to enhance comparability, the association was by far strongest for MRscore and cont.MRscore, followed by FI and AA. Including all 3 indicators of biological aging in the same model did not substantially attenuate risk estimates for cont.MRscore and FI, but HRs for AA were strongly attenuated and no longer statistically significant. All those findings were confirmed in subset III.

**Table 3:** Associations of methylomic survival predictors and frailty index with all-cause mortality.

Predictor	Subset I + subset II (n=1851; meta-analysis)				Subset III (n=470)		
	HR (95% CI)				OR (95% CI)		
	Deaths	Model 1^a^	Model 2^b^		Deaths	Model 1^a^	Model 2^b^
MRscore^c^ = 0	49	Ref	Ref		4	Ref	Ref
MRscore^c^ = 1	100	1.65 (1.14 – 2.40)	1.66 (1.13 – 2.42)		10	Ref	Ref
MRscore^c^ = 2-5	348	2.23 (1.59 – 3.13)	2.03 (1.43 – 2.88)		83	1.49 (0.69 – 3.22)	1.57 (0.72 – 3.43)
MRscore^c^ = >5	200	4.46 (2.96 – 6.73)	3.70 (2.42 – 5.64)		102	3.97 (1.62 – 9.70)	3.81 (1.53 – 9.47)
							
cont.MRscore^d^ (per 1 unit)	697	4.44 (3.22 – 6.11)	3.86 (2.77 – 5.31)		199	7.29 (3.30 – 16.10)	6.71 (2.99 – 15.05)
Age acceleration^e^ (per 5-years)	697	1.16 (1.00 – 1.35)	1.05 (0.90 – 1.22)		199	1.22 (0.97 – 1.54)	1.09 (0.86 – 1.40)
Frailty index (per 10%)	697	1.27 (1.19 – 1.35)	1.24 (1.16 – 1.32)		199	1.21 (1.05 – 1.38)	1.18 (1.02 – 1.36)
							
MRscore^c^ (per SD)	697	1.62 (1.45 – 1.82)	1.55 (1.35 – 1.74)		199	1.96 (1.44 – 2.66)	1.91 (1.39 – 2.62)
							
cont.MRscore^d^ (per SD)	697	2.04 (1.75 – 2.37)	1.91 (1.63 – 2.22)		199	2.65 (1.80 – 3.90)	2.54 (1.71 – 3.77)
Age acceleration^e^ (per SD)	697	1.16 (1.00 – 1.35)	1.05 (0.90 – 1.22)		199	1.21 (0.97 – 1.52)	1.07 (0.84 – 1.36)
Frailty index (per SD)	697	1.42 (1.30 – 1.55)	1.37 (1.25 – 1.51)		199	1.34 (1.08 – 1.67)	1.29 (1.02 – 1.62)

Abbreviations: CI, confidence interval; cont.MRscore, continuous mortality risk score; HR, hazard ratio; MRscore, mortality risk score; Ref., reference category.

^a^Model 1: adjusted for age, sex, leukocyte composition (not for FI), smoking status and alcohol consumption; ^b^Model 2: like model 1, results for MRscore/cont.MRscore additionally adjusted for epigenetic age acceleration and frailty index, results for epigenetic age acceleration, additionally adjusted for cont.MRscore and frailty index, and results for frailty index, additionally adjusted for cont.MRscore and epigenetic age acceleration; ^c^MRscore based on aberrant methylation of 10 CpGs (cg01612140, cg05575921, cg06126421, cg08362785, cg10321156, cg14975410, cg19572487, cg23665802, cg24704287, cg25983901): 0-10 refer to simultaneously aberrant methylation at 0 to 10 CpGs. ^d^cont.MRscore refers to a risk score computed through linear combination of weighted methylation values of the 10 CpGs. ^e^Age acceleration estimated by the residuals of DNA methylation age (estimated using Horvarth’s algorithm) regressed on chronological age.

Figure 2a presents prediction error curves of these models in the analysis of subset I. From 9-year survival to 14-year survival, the prediction error calculated by FI was smaller than by AA, and larger than by MRscore. Combining FI and MRscore reduced the prediction error further, but further combination with AA did not improve the prediction accuracy (its prediction error curve overlapped with the curve for FI and MRscore combined). A similar pattern of prediction error curves was also observed in subset II (Figure 2b, curves can only be plotted in the subcohort of subset II because of the case-cohort design). Table 4 presents Harrell’s C that provides a global assessment of a fitted survival model. Cont.MRscore outperformed AA [C-statistics, 0.676 (0.644 – 0.709) versus 0.535 (0.500 – 0.570), p-value<0.0001] and FI [C-statistics, 0.676 (0.644 – 0.709) versus 0.626 (0.591 – 0.659), p-value=0.02] in subset I, and even better performance was observed when combining cont.MRscore and FI [C-statistics, 0.705 (0.673 – 0.737)], and performance improved further by adding chronological age in the model [C-statistics, 0.740 (0.709 – 0.770)]. Consistent patterns were also demonstrated when estimating time-dependent AUCs (Figure 2c and 2d). Similar results were also obtained in the subcohort samples of subset II (Table 4, Figure 2e and 2f) and in subset III (Table 4, Figure 2g and 2h).

**Figure 2a-d:**
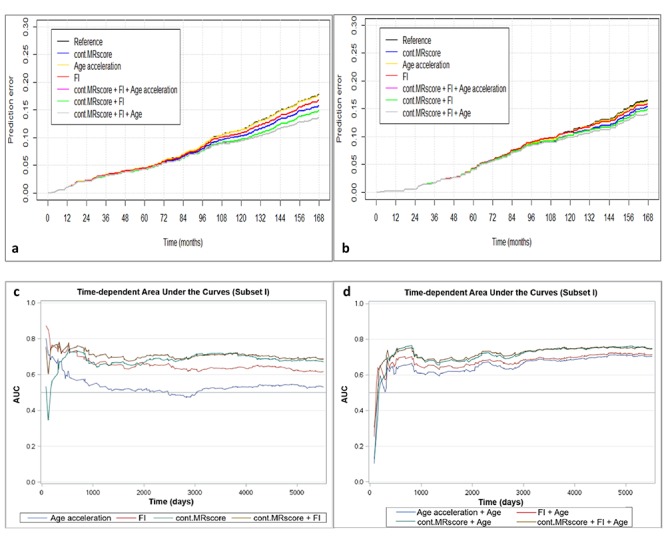
**Predictive performance of methylomic survival predictors and frailty index.** (**a**) predictive prediction error curves in subset I; (**b**) predictive prediction error curves in subcohort of subset II; (**c**) Time-dependent area under the curves (AUCs) of methylomic survival predictors and frailty index in subset I; (**d**) Time-dependent AUCs of combination of age with methylomic survival predictors and frailty index in subset I.

**Table 4:** Harrell's C statistics of chronological age, methylomic survival predictors, and frailty in prediction of mortality.

Predictor	Harrell's C statistics (95% CI)			C-index (logistic regression)
	Subset I	Subset II		Subset III
Age	0.675 (0.643 – 0.707)	0.659 (0.612 – 0.707)		0.586 (0.535 – 0.638)
DNA methylation age	0.659 (0.626 – 0.691)	0.654 (0.607 – 0.701)		0.613 (0.561 – 0.665)
DNA methylation age + age	0.679 (0.647 – 0.711)	0.667 (0.620 – 0.714)		0.614 (0.563 – 0.667)
Age acceleration	0.535 (0.500 – 0.570)	0.548 (0.497 – 0.599)		0.581 (0.529 – 0.634)
Age acceleration + age	0.679 (0.647 – 0.711)	0.667 (0.620 – 0.714)		0.614 (0.563 – 0.667)
cont.MRscore	0.676 (0.644 – 0.709)	0.656 (0.606 – 0.705)		0.715 (0.669 – 0.762)
cont.MRscore + age	0.740 (0.709 – 0.770)	0.706 (0.661 – 0.752)		0.725 (0.680 – 0.771)
Frailty index	0.626 (0.591 – 0.659)	0.626 (0.580 – 0.672)		0.609 (0.557 – 0.661)
Frailty index + age	0.693 (0.662 – 0.724)	0.684 (0.639 – 0.728)		0.634 (0.583 – 0.684)
cont.MRscore + Frailty index	0.705 (0.673 – 0.737)	0.681 (0.635 – 0.727)		0.725 (0.679 – 0.770)
cont.MRscore + Frailty index + age	0.748 (0.718 – 0.777)	0.718 (0.675 – 0.762)		0.732 (0.687 – 0.777)

Abbreviations: cont.MRscore, continuous mortality risk score.

**Figure 2e-h:**
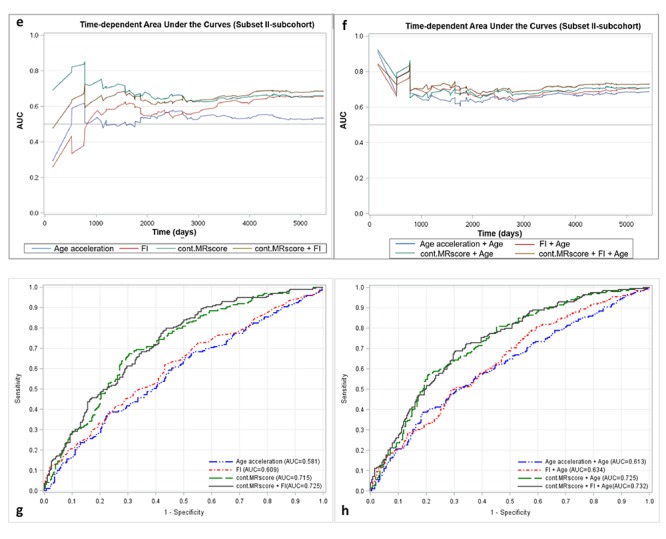
**Predictive performance of methylomic survival predictors and frailty index.** (**e**) time-dependent AUCs of methylomic survival predictors and frailty index in subcohort of subset II; (f) time-dependent AUCs of combination of age with methylomic survival predictors and frailty index in subcohort of subset II; (**g**) AUCs of methylomic survival predictors and frailty index in subset III; (**h**) AUCs of combination of age with methylomic survival predictors and frailty index in subset III.

## DISCUSSION

In this study of more than 2300 community-dwelling older adults with 14 years of follow-up, we demonstrated that our newly derived MRscore was strongly associated with frailty estimated by accumulation of 34 health deficits. The association was much stronger compared to that between frailty and the other methylomic survival predictor, the epigenetic clock-derived AA. The MRscore predicted all-cause mortality better than FI, a well-established measure of frailty. Survival prediction was improved by combining MRscore and FI, whereas the epigenetic AA had no independent predictive value in models containing MRscore and FI. These findings were validated in samples that did not overlap with samples from which the MRscore was derived, and demonstrated the ability of the MRscore to strongly enhance survival prediction beyond established markers of biological age, such as FI and AA.

The MRscore was derived from an epigenome-wide screening for mortality-related DNA methylation changes [18]. It exhibited strikingly strong associations with mortality outcomes, compared to those of common environmental, molecular, and genetic risk factors [19, 20]. In the current study, we further verified it as a survival predictor via its strong association with frailty, a well-defined syndrome that goes along with an increased risk of death [6]. Frailty is caused by aging-related decline in reserve and function across multiple physiologic systems, such as impairments in immune/inflammatory [21, 22], neuromuscular deregulations [23], metabolic and vascular alterations [24, 25], and oxidative stress [26]. Frail individuals are thus characterized by increased vulnerability to age-related disorders, such as myocardial infarction, rheumatoid arthritis, diabetes, hypertension, and cognitive impairments [27, 28]. The observed association between MRscore and FI was therefore not unexpected. Of 10 CpGs included in the MRscore, 6 CpGs map to intergenic regions with unknown function, and the other 4 CpGs are annotated to genes involved in common chronic disease, including atherosclerosis, myocardial infarction, and multiple types of cancers [18, 29-32]. The shared linkage with morbidity may therefore explain the association between MRscore and FI. However, due to the cross-sectional nature of the analyses of their association, any inferences regarding a potential causal relationship between both indicators cannot be drawn. On the other hand, the independent predictive capacities for mortality of both indicators demonstrated in the current study suggest that they at least partly reflect different, complementary pathophysiological pathways leading to fatal outcomes. In addition, in the current study we also observed associations with FI for many other mortality-related CpGs, some of which showed even stronger associations than the 10 CpGs used to compute the MRscore. Future studies with longitudinal data of both methylation profiles and FI are needed to provide a clearer picture of the development of methylation and frailty changes, as well as their roles in aging-related phenotypes including mortality.

Survival predictors reflecting individuals’ biological age with high accuracy bear clinical applications for identifying people at high risk and tailoring healthcare, and also are of paramount importance to research on human aging. The survival predictors can serve as surrogate endpoints for studies that may otherwise last decades and require much greater resources [33, 34]. For instance, clinical trials evaluating drugs or therapeutic approaches that aim to counteract aging related endpoints such as mortality theoretically need lifespan observations to determine effects. With use of reliable survival predictors as surrogate endpoints, such clinical trials would benefit greatly in terms of duration and expense. Various drugs, such as metformin, acarbose, angiotensin receptor blockers, and rapamycin, have shown protective effects with respect to age-related health deteriorations in mouse models [35-37]. Our MRscore, its combination with FI, or the combination of both indicators with other powerful predictors could facilitate moving such promising drugs or therapies into clinical trials in the future. Likewise, the MRscore could also be a useful tool to facilitate evaluation of other types of health intervention and promotion. Currently, multiple medical prevention platforms, such as smart phone-based instruments, are being established to promote health habits and postpone aging-related health decline. A reliable and objective indicator of longevity like the MRscore might help to motivate and guide subjects to adhere to the active intervention in such a context. However, further evidence, on the dynamic changes of the MRscore in response to lifestyle changing or intervention, and on the biological significance of the DNAm markers (of the MRsocre) in relation to diseases, is needed as a basis for implementing the MRscore as surrogate endpoint in clinical practice.

The epigenetic clock derived AA is a recently established survival predictor. It has been linked to a broad range of aging-related phenotypes, including Werner syndrome [38], physical fitness [39], cognitive functioning [13, 40], immunological disorders [41], coronary heart disease [42], and various forms of cancer, such as lung, breast, and colorectal cancer [12, 14]. The association of AA with two other survival predictors, the MRscore and FI, shown in the current study supports the idea that this indicator reflects biological age to some extent. However, its association with mortality disappeared after adjustment for MRscore and FI, which is consistent with findings from a previous study by Kim and colleagues that estimated FI and AA together and showed the association with mortality only for FI [17]. The authors concluded that small effects of the DNAm age or AA on survival require large samples to be detected, and DNAm age or AA might largely be a statistical reflection of effects of chronological age. In fact DNAm age was initially trained as precisely as possible to predict chronological age. Here we also showed that the predictive accuracy of DNAm age for mortality is similar as of chronological age (Table 4), and model fit was improved marginally when combining chronological age with either DNAm age or AA, yielding the same C-statistics, of note. By contrast, MRscore and FI were confirmed to be highly predictive survival indicators beyond chronological age.

The population-based cohort study design, long-term mortality follow-up, comprehensive collection of health data, side by side comparison of the 3 survival predictors in the same study population, meta-analyzing data according to methylation experiment batch, and validation in independent samples are major strengths of the current study. On the other hand, several limitations have to be addressed. The cross-sectional analysis on the association between mortality-related methylation markers and frailty prohibits any conclusions as to the temporality and causality of their relationships. In addition, potential overestimation of MRscore in prediction of mortality may exist, given that the MRscore was initially derived from the ESTHER study population. However, the MRscore has been independently verified in another population-based cohort from Germany, where the MRscore exhibited equally predictive capacity as in the current study [18]. Moreover, in the current study we yielded consistent findings in independent ESTHER samples which had not been included in the derivation of the MRscore, suggesting that potential overestimation of predictive capacity is likely to be small.

Given an aging population worldwide, a reliable survival predictor is highly desirable and bears applications in the clinical, public health, and research fields. Our MRscore may serve as a good candidate in this respect, and its combination with other robust survival predictors to enhance prediction of aging-related phenotypes as illustrated for the combination with FI in the present study warrants further exploration in future studies.

## METHODS

### Study population and data collection

The study population consisted of three subsets of participants from the ESTHER cohort, a population-based epidemiological study conducted in Southwest Germany. Details of the study population have been described previously [18]. In brief, among 9,949 participants (age 50-75 years) recruited in the ESTHER study at baseline (between 2000 and 2002), three subsets were selected for DNAm assessment (Supplementary Figure S1): Subset I consists of 1,000 participants consecutively enrolled during the first 6 months of recruitment; Subset II consists of 864 participants selected for a case-cohort design for mortality analysis [18]; Subset III, which was primarily selected to address cancer-related methylation signatures, consists of 266 participants who had a first diagnosis of any of 3 types of cancer (i.e. lung, colorectal, and head-and-neck cancer) during 14 years of follow-up and were not included in the Subset I and II, and 205 participants randomly selected among those free from the 3 types of cancer by the end of 14-year follow-up. During the baseline enrollment, epidemiological data, including socio-demographic characteristics, lifestyle factors, and medical history, were collected via a standardized self-administered questionnaire completed by participants and via additional reports from participants’ general practitioners, and biological samples (blood, stool, urine) were obtained and stored at −80 °C. Vital status was followed up through record linkage with population registries in Saarland until December 31, 2015. The study was approved by the ethics committees of the University of Heidelberg and of the Medical Association of Saarland. All participants provided written informed consent.

### Methylation assessment

DNAm in baseline blood samples was determined using the Infinium HumanMethylation450K BeadChip Assay (Illumina.Inc, San Diego, CA, USA). Methodological details have been reported previously [31]. Data were normalized by pre-processing in GenomeStudio. In addition, probes with detection p-value>0.01, with missing values>10%, and targeting the X and Y chromosomes were excluded in data pre-processing. Methylation beta values of 58 mortality-related CpGs were extracted. The epigenetic clock, estimated by Hovarth’s DNAm age [8], was calculated using the online tool available at https://dnamage.genetics.ucla.edu/.

### Frailty index

The FI, calculated as previously described [6], quantifies the ratio of deficits presented over the total number of deficits considered. The deficits in health refer to multiple types of symptoms, signs, disabilities, diseases, or aberrance of biomarkers. In the ESTHER study, following a standard procedure of the deficits selection and FI construction, a FI was calculated based on 34 deficits that were associated with the general health status, accumulated with age, did not saturate too early, had more than 1% prevalence, and did not have a high prevalence (>50%) at younger ages (50-60 years) [6]. The list of deficits included in the FI is provided in Supplementary Table S1. Missing values in the variables used to calculate FI were taken care of by multiple imputation using the SAS procedure PROC MI, and regression results for FI in the present analysis were based on 20 imputations combined by the SAS procedure MIANALYZE.

### Statistical analysis

Descriptive analyses were first performed to explore the distribution and mutual correlations of chronological age, DNAm age, AA (calculated as residuals of Horvarth’s DNAm age regressed on chronological age), MRscore, and FI using histograms, scatter plots, and Spearman correlation coefficients in the three subsets of the study population separately. The mortality risk score was constructed in two forms as in the previous study [18]: MRscore was constructed according to aberrant methylation of 10 CpGs (cg01612140, cg05575921, cg06126421, cg08362785, cg10321156, cg14975410, cg19572487, cg23665802, cg24704287, cg25983901) and ranges from 0 (aberrant methylation at none of the 10 CpGs) to10 (aberrant methylation at all 10 CpGs) [aberrance was defined by the highest quartile value for the hypermethylated CpG (cg08362785) and by the lowest quartile values for the hypomethylated CpGs (other 9 CpGs) from the previous study [18]]; a continuous mortality risk score (cont.MRscore) was computed by summarizing weighted methylation β values of the 10 CpGs [cg01612140*(-0.38253) + cg05575921*(-0.92224) + cg06126421*(-1.70129) + cg08362785*(2.71749) + cg10321156*(-0.02073) + cg14975410* (-0.04156) + cg19572487*(-0.28069) + cg23665802*(-0.89440) + cg24704287*(-2.98637) + cg25983901*(-1.80325); weights were derived from the least absolute shrinkage and selection operator (LASSO) regression from the previous study [18].

#### Associations of MRscore/cont.MRscore and age acceleration with FI

The individual and joint associations of MRscore/cont.MRscore and epigenetic AA with FI were assessed by mixed linear regression models, with batch as random effect. Models were first adjusted for chronological age, sex, and leukocyte composition estimated using Houseman’s algorithm [43] (Model 1), and then additionally adjusted for smoking status and alcohol consumption (Model 2). The analyses were first carried out in subset I, II, and III separately, and then summarized by random effects meta-analysis (Supplementary Figure S1). To further assess the relationship between mortality related DNAm changes and frailty, the associations between individual 58 CpGs identified in our previous study [18] and FI were also analyzed by mixed linear regression models as described above. Multiple testing was corrected for using the Benjamini-Hochberg approach (FDR<0.05) in the meta-analysis of the three subsets.

#### Associations of MRscore/cont.MRscore, age acceleration, and FI with all-cause mortality

To examine the individual and joint values of MRscore/cont.MRscore, AA, and FI in prediction of all-cause mortality, multivariate Cox regression models were fitted in subset I. In subset II, modified weighted Cox regression models were applied accounting for over-sampling deaths in the case-cohort design as described in the previous study (weight=1 / subcohort sampling fraction) [18]. Hazard ratios (HR) and 95% CIs were estimated for categorized MRscore (score = 0/1/2-5/5+), per 1 unit of the cont.MRscore, per 5-year AA, per 10% units FI, and also for per standard deviation (SD) increase in each predictor. In subset III, which had a nested case-control study design, multivariate logistic regression models were fitted, and odds ratios (OR) and 95% CIs were estimated correspondingly. Given that the MRscore/cont.MRscore were derived from the subset I and II and also the different design of subset III compared to subset I and II, random effects meta-analysis was utilized for combining results from the subsets I and II, and subset III served as a validation samples (Supplementary Figure S1). To assess the predictive accuracy of these survival indicators and their combination, and also the joint predictive power along with chronological age, 3 types of measures, i.e. prediction error curves, Harrell’s C-statistics, and time-dependent areas under the curve (AUCs), were additionally calculated in subset I and II. C-index and receiver operating characteristic (ROC) curves were calculated in subset III. Prediction error curves were plotted using the R package ‘pec’, all other statistical analyses were carried out in SAS 9.4 (SAS Institute, Cary, NC).

## Supplementary Material

Supplementary File
