# Supplementary material for: Methylomic survival predictors, frailty, and mortality
Source: Aging (Albany NY). 2018 Mar 6;10(3):339–57. doi: 10.18632/aging.101392 (PMC5892685; doi:10.18632/aging.101392)
Supplement: Supplementary File [file aging-10-101392-s001.pdf]

SUPPLEMENTARY MATERIAL

Table S1. Deficits included in the frailty index.

Deficits	
• **poor self-rated general health**	
• **history of various diseases (11 items)**	myocardial infarction<br>angina pectoris<br>heart failure<br>stroke<br>hypertension<br>hyperlipidemia<br>diabetes<br>cataract<br>glaucoma<br>gout<br>cancer
• **difficulties in the activities of daily living (16 items)**	"vigorous activities"<br>"climbing several flights of stairs"<br>"climbing one flight of stairs"<br>"walking more than one mile"<br>"walking several blocks"<br>"walking one block"<br>"moderate activities, such as moving a table, pushing a vacuum cleaner, bowling, or playing golf"<br>"lifting or carrying groceries"<br>"bathing or dressing yourself"<br>"bending, kneeling or stooping"<br>"limits in normal work or activities due to pain"<br>"accomplished less work or activities due to impaired physical health"<br>"limits in type of work or activities due to impaired physical health"<br>"difficulties chewing hard food"<br>"difficulties chewing meat"<br>"short-term memory loss"
• **symptoms (6 items)**	under-/overweight<br>pyrosis<br>shiver<br>insomnia<br>costiveness<br>aconuresis

Table S2. Associations between individual mortality-related CpGs and frailty index.

CpG sites	Gene name	Subset I (n=993)			Subset II (n=858)			Subset III (n=470)			Overall (meta-analysis)			
		Coefficient (95% CI)[a]		p-value	Coefficient (95% CI)[a]		p-value	Coefficient (95% CI)[a]		p-value	Coefficient (95% CI)[a]		p-value	FDR
cg01406381	*SLC1A5*	-10.03	(-13.84, -6.22)	2.43E-07	-8.04	(-12.09, -4.00)	9.75E-05	-12.83	(-20.15, -5.50)	0.0006	-9.56	(-12.16, -6.97)	4.89E-13	2.84E-11
cg07626482	*SLC1A5*	-6.90	(-10.26, -3.54)	5.79E-05	-7.97	(-11.52, -4.41)	1.14E-05	-6.05	(-10.70, -1.40)	0.0108	-7.11	(-9.27, -4.95)	1.18E-10	3.42E-09
cg19859270	*GPR15*	-9.76	(-14.83, -4.68)	0.0002	-8.66	(-13.21, -4.11)	0.0002	-5.63	(-12.39, 1.12)	0.1022	-8.44	(-11.47, -5.41)	4.67E-08	9.02E-07
cg19266329		-6.01	(-8.78, -3.25)	2.00E-05	-3.61	(-6.46, -0.76)	0.013	-4.70	(-9.13, -0.27)	0.0375	-4.82	(-6.63, -3.01)	1.76E-07	2.56E-06
cg25607249	*SLC1A5*	-6.44	(-10.25, -2.64)	0.0009	-6.50	(-10.54, -2.46)	0.0016	-5.72	(-13.04, 1.60)	0.1255	-6.38	(-8.97, -3.78)	1.42E-06	1.65E-05
cg12510708	*NFE2L3*	-5.46	(-8.13, -2.78)	6.45E-05	-2.98	(-5.72, -0.24)	0.0328	-4.18	(-8.78, 0.43)	0.0756	-4.24	(-6.00, -2.47)	2.63E-06	2.18E-05
cg23842572	*MPRIP*	6.08	(3.03, 9.13)	9.23E-05	4.21	(1.11, 7.32)	0.0077	2.85	(-2.61, 8.30)	0.3062	4.85	(2.83, 6.86)	2.55E-06	2.18E-05
cg03725309	*SARS*	-6.47	(-9.71, -3.23)	8.97E-05	-4.36	(-7.70, -1.03)	0.0104	-2.01	(-7.74, 3.73)	0.4930	-4.96	(-7.12, -2.81)	6.29E-06	4.56E-05
cg24704287		-3.43	(-5.80, -1.05)	0.0047	-3.54	(-6.20, -0.88)	0.009	-4.73	(-8.82, -0.64)	0.0235	-3.68	(-5.30, -2.05)	9.32E-06	5.40E-05
cg25189904	*GNG12*	-1.87	(-3.10, -0.64)	0.0029	-1.57	(-2.96, -0.17)	0.0275	-2.62	(-4.58, -0.66)	0.0088	-1.90	(-2.73, -1.06)	8.49E-06	5.40E-05
cg23190089	*SLC22A18AS*	-8.24	(-12.96, -3.53)	0.0006	-5.78	(-10.50, -1.06)	0.0165	-6.91	(-15.85, 2.04)	0.1303	-7.00	(-10.13, -3.87)	1.14E-05	6.00E-05
cg05492306	*ERCC1*	-6.96	(-10.46, -3.45)	9.95E-05	-3.27	(-6.69, 0.15)	0.0609	-6.89	(-12.52, -1.25)	0.0166	-5.41	(-7.98, -2.85)	3.60E-05	0.0002
cg04987734	*CDC42BPB*	2.98	(0.70, 5.27)	0.0105	2.51	(0.11, 4.90)	0.0403	5.45	(1.56, 9.33)	0.0060	3.17	(1.65, 4.69)	4.47E-05	0.0002
cg14975410		-3.18	(-5.08, -1.29)	0.001	-1.88	(-3.93, 0.17)	0.0726	-2.63	(-5.52, 0.26)	0.0743	-2.59	(-3.85, -1.34)	5.13E-05	0.0002
cg03636183	*F2RL3*	-2.25	(-3.97, -0.52)	0.0106	-2.30	(-4.06, -0.53)	0.0108	-1.84	(-4.23, 0.55)	0.1314	-2.18	(-3.28, -1.09)	9.62E-05	0.0004
cg26470501	*BCL3*	-2.80	(-5.36, -0.24)	0.0321	-3.57	(-6.28, -0.86)	0.0098	-3.76	(-7.74, 0.21)	0.0635	-3.27	(-4.96, -1.59)	0.0001	0.0005
cg05575921	*AHRR*	-1.60	(-2.82, -0.38)	0.0102	-1.78	(-3.02, -0.54)	0.005	-0.52	(-2.40, 1.35)	0.5848	-1.48	(-2.27, -0.69)	0.0002	0.0008
cg07986378	*ETV6*	-3.68	(-5.90, -1.46)	0.0011	-1.72	(-4.10, 0.65)	0.1551	-2.87	(-6.66, 0.92)	0.1377	-2.78	(-4.27, -1.29)	0.0002	0.0008
cg06126421		-1.53	(-3.04, -0.02)	0.0465	-3.04	(-4.56, -1.51)	9.34E-05	-1.19	(-3.39, 1.01)	0.2892	-2.04	(-3.17, -0.91)	0.0003	0.0011
cg08362785	*MKL1*	5.83	(2.10, 9.56)	0.0022	3.82	(-0.34, 7.98)	0.0721	2.84	(-3.39, 9.07)	0.3722	4.59	(2.05, 7.13)	0.0004	0.0011
cg18181703	*SOCS3*	-5.16	(-7.47, -2.85)	1.18E-05	-4.41	(-6.90, -1.93)	0.0005	-1.03	(-4.50, 2.43)	0.5592	-3.86	(-6.03, -1.68)	0.0005	0.0014
cg00310412	*SEMA7A*	-5.64	(-8.44, -2.84)	7.82E-05	-2.11	(-5.20, 0.98)	0.1805	-4.53	(-9.36, 0.30)	0.0661	-4.09	(-6.41, -1.77)	0.0005	0.0014
cg23665802	*MIR19A*	-4.39	(-7.08, -1.69)	0.0014	-1.81	(-4.44, 0.82)	0.1765	-2.45	(-6.77, 1.88)	0.2671	-2.97	(-4.69, -1.24)	0.0007	0.0019
cg02657160	*CPOX*	-4.87	(-8.60, -1.14)	0.0105	-2.38	(-5.99, 1.24)	0.1976	-6.47	(-12.30, -0.64)	0.0297	-4.06	(-6.43, -1.69)	0.0008	0.0019
cg19572487	*RARA*	-4.04	(-6.08, -2.01)	9.76E-05	-1.70	(-3.97, 0.57)	0.1422	-1.81	(-5.08, 1.45)	0.2768	-2.72	(-4.35, -1.10)	0.0010	0.0024
cg01572694	*MIR10A*	-2.38	(-4.63, -0.13)	0.0381	-2.61	(-4.99, -0.23)	0.0317	-2.47	(-6.29, 1.35)	0.2050	-2.49	(-3.99, -0.98)	0.0012	0.0027
cg12513616		-3.15	(-5.81, -0.49)	0.0204	-3.39	(-6.42, -0.35)	0.0287	-0.89	(-5.10, 3.32)	0.6792	-2.82	(-4.62, -1.01)	0.0023	0.0048
cg26709988	*CRISPLD2*	-3.40	(-6.38, -0.42)	0.0256	-1.96	(-5.15, 1.23)	0.2278	-4.14	(-10.23, 1.95)	0.1824	-2.89	(-4.94, -0.84)	0.0058	0.0120
cg15342087		-2.32	(-5.60, 0.96)	0.1658	-4.83	(-7.99, -1.66)	0.0028	-0.59	(-5.79, 4.61)	0.8250	-3.08	(-5.33, -0.84)	0.0071	0.0141

Table S2. continued.

CpG sites	Gene name	Subset I (n=993)		Subset II (n=858)		Subset III (n=470)		Overall (meta-analysis)		
		Coefficient (95% CI)[a]	p-value	Coefficient (95% CI)[a]	p-value	Coefficient (95% CI)[a]	p-value	Coefficient (95% CI)[a]	p-value	FDR
cg25983901		-2.46 (-4.69, -0.22)	0.0310	-2.01 (-4.28, 0.26)	0.0826	0.13 (-3.24, 3.50)	0.9390	-1.81 (-3.24, -0.37)	0.0139	0.0269
cg03707168	*PPP1R15A*	-2.50 (-5.07, 0.06)	0.0555	-1.85 (-4.47, 0.76)	0.1643	-1.18 (-5.68, 3.33)	0.6091	-2.04 (-3.74, -0.35)	0.0182	0.0341
cg26963277	*KCNQ1OT1*	-4.43 (-7.08, -1.78)	0.0011	-0.80 (-3.48, 1.87)	0.5549	-3.12 (-7.09, 0.84)	0.1220	-2.74 (-5.09, -0.40)	0.0218	0.0394
cg06905155		-1.63 (-4.21, 0.94)	0.2139	-2.46 (-5.47, 0.55)	0.1087	-2.22 (-6.48, 2.04)	0.3066	-2.03 (-3.80, -0.25)	0.0256	0.0449
cg18550212	*ATL3*	-4.43 (-7.24, -1.61)	0.0020	-2.57 (-5.59, 0.45)	0.0951	0.66 (-4.41, 5.74)	0.7980	-2.75 (-5.21, -0.29)	0.0284	0.0484
cg24397007	*FOSL2*	-1.79 (-4.40, 0.83)	0.1802	-1.40 (-4.21, 1.41)	0.3280	-3.57 (-7.91, 0.76)	0.1064	-1.93 (-3.68, -0.18)	0.0308	0.0511
cg07123182	*KCNQ1OT1*	-4.15 (-8.55, 0.25)	0.0645	-1.44 (-5.53, 2.66)	0.4910	-3.68 (-9.42, 2.05)	0.2081	-2.91 (-5.56, -0.25)	0.0319	0.0513
cg11341610	*CALR*	-2.77 (-6.07, 0.53)	0.0996	-2.18 (-5.47, 1.12)	0.1957	-0.82 (-6.66, 5.02)	0.7830	-2.25 (-4.41, -0.08)	0.0420	0.0641
cg23079012		-2.86 (-6.05, 0.34)	0.0794	-1.71 (-4.11, 0.69)	0.1624	-0.55 (-3.74, 2.64)	0.7348	-1.71 (-3.35, -0.06)	0.0419	0.0641
cg01140244	*INPP5A*	2.56 (-1.94, 7.05)	0.2650	3.97 (-0.59, 8.53)	0.0881	1.67 (-6.92, 10.26)	0.7024	3.06 (0.06, 6.06)	0.0456	0.0664
cg27241845		-3.88 (-5.87, -1.89)	0.0001	-1.81 (-3.88, 0.26)	0.0873	-0.10 (-2.92, 2.73)	0.9460	-2.11 (-4.17, -0.04)	0.0458	0.0664
cg08546016	*TMEM104*	-2.91 (-5.94, 0.12)	0.0594	-3.83 (-6.86, -0.81)	0.0130	2.29 (-3.81, 8.39)	0.4614	-2.48 (-5.15, 0.19)	0.0691	0.0977
cg14817490	*AHRR*	-1.78 (-4.00, 0.43)	0.1149	-1.82 (-4.08, 0.44)	0.1147	0.87 (-2.47, 4.21)	0.6111	-1.31 (-2.74, 0.12)	0.0725	0.1001
cg15459165	*LAPTM5*	3.14 (-0.50, 6.78)	0.0913	1.17 (-2.30, 4.64)	0.5098	1.55 (-4.38, 7.48)	0.6084	2.02 (-0.29, 4.33)	0.0869	0.1172
cg26286961	*CSGALNACT1*	-1.88 (-3.76, -0.01)	0.0484	-0.44 (-2.17, 1.30)	0.6197	-0.22 (-2.86, 2.42)	0.8708	-0.94 (-2.09, 0.21)	0.1079	0.1423
cg13854219		-8.14 (-11.80, -4.48)	1.32E-05	-1.25 (-4.82, 2.31)	0.4916	-0.59 (-6.38, 5.20)	0.8418	-3.54 (-8.57, 1.49)	0.1683	0.2122
cg19459791		4.00 (1.57, 6.42)	0.0012	0.18 (-2.58, 2.94)	0.9001	0.83 (-3.25, 4.92)	0.6890	1.85 (-0.78, 4.47)	0.1677	0.2122
cg25491402	*PDE9A*	0.19 (-3.98, 4.37)	0.9278	2.63 (-1.65, 6.90)	0.2285	5.29 (-2.14, 12.72)	0.1630	1.93 (-0.85, 4.70)	0.1735	0.2141
cg00285394	*SQLE*	-2.86 (-4.57, -1.14)	0.0011	-0.91 (-2.86, 1.04)	0.3582	0.55 (-2.22, 3.32)	0.6972	-1.29 (-3.18, 0.59)	0.1787	0.2159
cg16503724	*PLCL2*	-1.67 (-5.22, 1.88)	0.3566	-1.42 (-4.82, 1.98)	0.4128	-0.37 (-5.77, 5.04)	0.8943	-1.34 (-3.58, 0.90)	0.2404	0.2845
cg01612140		-4.08 (-6.72, -1.45)	0.0024	-0.69 (-3.27, 1.90)	0.6025	0.98 (-3.50, 5.46)	0.6673	-1.58 (-4.44, 1.28)	0.2779	0.3161
cg14085840		-5.56 (-9.77, -1.36)	0.0095	-1.48 (-5.22, 2.26)	0.4392	2.11 (-4.53, 8.74)	0.5337	-2.18 (-6.08, 1.73)	0.2745	0.3161
cg21161138	*AHRR*	-3.94 (-6.32, -1.56)	0.0012	-2.99 (-5.51, -0.46)	0.0204	2.75 (-0.68, 6.19)	0.1162	-1.59 (-5.20, 2.03)	0.3895	0.4344
cg10321156		-2.09 (-3.57, -0.61)	0.0058	0.58 (-0.96, 2.12)	0.4598	-0.81 (-3.49, 1.88)	0.5562	-0.78 (-2.60, 1.04)	0.4031	0.4411
cg25763716	*VCAM1*	-4.35 (-7.71, -0.99)	0.0112	0.87 (-1.89, 3.63)	0.5372	-0.94 (-6.89, 5.01)	0.7567	-1.45 (-5.09, 2.18)	0.4336	0.4657
cg14855367	*UTS2D*	-1.63 (-4.24, 0.99)	0.2231	-0.51 (-3.15, 2.14)	0.7069	1.52 (-2.18, 5.23)	0.4198	-0.55 (-2.21, 1.11)	0.5170	0.5452
cg20732076	*TRERF1*	-4.01 (-7.46, -0.57)	0.0225	1.47 (-1.96, 4.90)	0.4011	0.07 (-6.00, 6.15)	0.9813	-0.96 (-4.75, 2.83)	0.6196	0.6417
cg25193885	*SHANK2*	2.61 (-2.07, 7.28)	0.2743	-2.77 (-7.29, 1.74)	0.2283	5.20 (-2.65, 13.04)	0.1943	1.05 (-3.49, 5.59)	0.6495	0.6609
cg25285720	*HLA-DMA*	-2.79 (-8.95, 3.38)	0.3756	1.23 (-3.33, 5.79)	0.5968	5.11 (-3.40, 13.63)	0.2393	0.66 (-3.05, 4.36)	0.7288	0.7288

Abbreviations: CI, confidence interval; FDR, false discovery rate. [a]Model adjusted for age, sex, leukocyte composition, smoking status and alcohol consumption; estimated for changes (95% confidence interval) in frailty index expressed in % units per 10% units higher methylation level.

Abbreviations: CI, confidence interval; FDR, false discovery rate. [a]Model adjusted for age, sex, leukocyte composition, smoking status and alcohol consumption; estimated for changes (95% confidence interval) in frailty index expressed in % units per 10% units higher methylation level.

Table S3. Associations of methylomic survival predictors and frailty index with all-cause mortality in the subset I and subset II.

Predictor	HR (95% CI)			
	Subset I (n=993)		Subset II (n=858)	
	Model 1[a]	Model 2[b]	Model 1[a]	Model 2[b]
MRscore[c] = 0	Ref	Ref	Ref	Ref
MRscore[c] = 1	1.52 (0.90 – 2.55)	1.47 (0.88 – 2.48)	1.71 (1.01 – 2.90)	1.80 (1.04 – 3.11)
MRscore[c] = 2-5	2.36 (1.45 – 3.84)	2.19 (1.33 – 3.58)	2.13 (1.35 – 3.36)	1.95 (1.21 – 3.14)
MRscore[c] = >5	5.34 (3.01 – 9.48)	4.57 (2.54 – 8.20)	3.84 (2.25 – 6.56)	3.20 (1.85 – 5.53)
cont.MRscore[d] (per 1 unit)	3.86 (2.56 – 5.83)	3.51 (2.28 – 5.40)	5.38 (3.32 – 8.71)	4.35 (2.64 – 7.17)
Age acceleration[e] (per 5-years)	1.09 (0.95 – 1.24)	0.96 (0.84 – 1.11)	1.25 (1.10 – 1.41)	1.12 (0.99 – 1.27)
Frailty index (per 10%)	1.27 (1.17 – 1.38)	1.26 (1.16 – 1.38)	1.26 (1.15 – 1.38)	1.20 (1.09 – 1.32)
MRscore[c] (per SD)	1.65 (1.41 – 1.92)	1.58 (1.35 – 1.86)	1.59 (1.37 – 1.86)	1.52 (1.25 – 1.85)
cont.MRscore[d] (per SD)	1.92 (1.57 – 2.34)	1.83 (1.49 – 2.26)	2.31 (1.82 – 2.94)	2.08 (1.62 – 2.66)
Age acceleration[e] (per SD)	1.08 (0.95 – 1.23)	0.96 (0.84 – 1.10)	1.26 (1.11 – 1.43)	1.13 (0.99 – 1.28)
Frailty index (per SD)	1.42 (1.26 – 1.60)	1.42 (1.25 – 1.61)	1.41 (1.23 – 1.62)	1.31 (1.13 – 1.52)

Abbreviations: CI, confidence interval; cont.MRscore, continuous mortality risk score; HR, hazard ratio; MRscore, mortality risk score; Ref., reference category.

[a]Model 1: adjusted for age, sex, leukocyte composition (not for FI), smoking status and alcohol consumption; [b]Model 2: like model 1, results for MRscore/cont.MRscore additionally adjusted for epigenetic age acceleration and frailty index, results for epigenetic age acceleration, additionally adjusted for cont.MRscore and frailty index, and results for frailty index, additionally adjusted for cont.MRscore and epigenetic age acceleration; [c]MRscore based on aberrant methylation of 10 CpGs (cg01612140, cg05575921, cg06126421, cg08362785, cg10321156, cg14975410, cg19572487, cg23665802, cg24704287, cg25983901): 0-10 refer to simultaneously aberrant methylation at 0 to 10 CpGs. [d]cont.MRscore refers to a risk score computed through linear combination of weighted methylation values of the 10 CpGs. [e]Age acceleration estimated by the residuals of DNA methylation age (estimated using Horvarth's algorithm) regressed on chronological age.

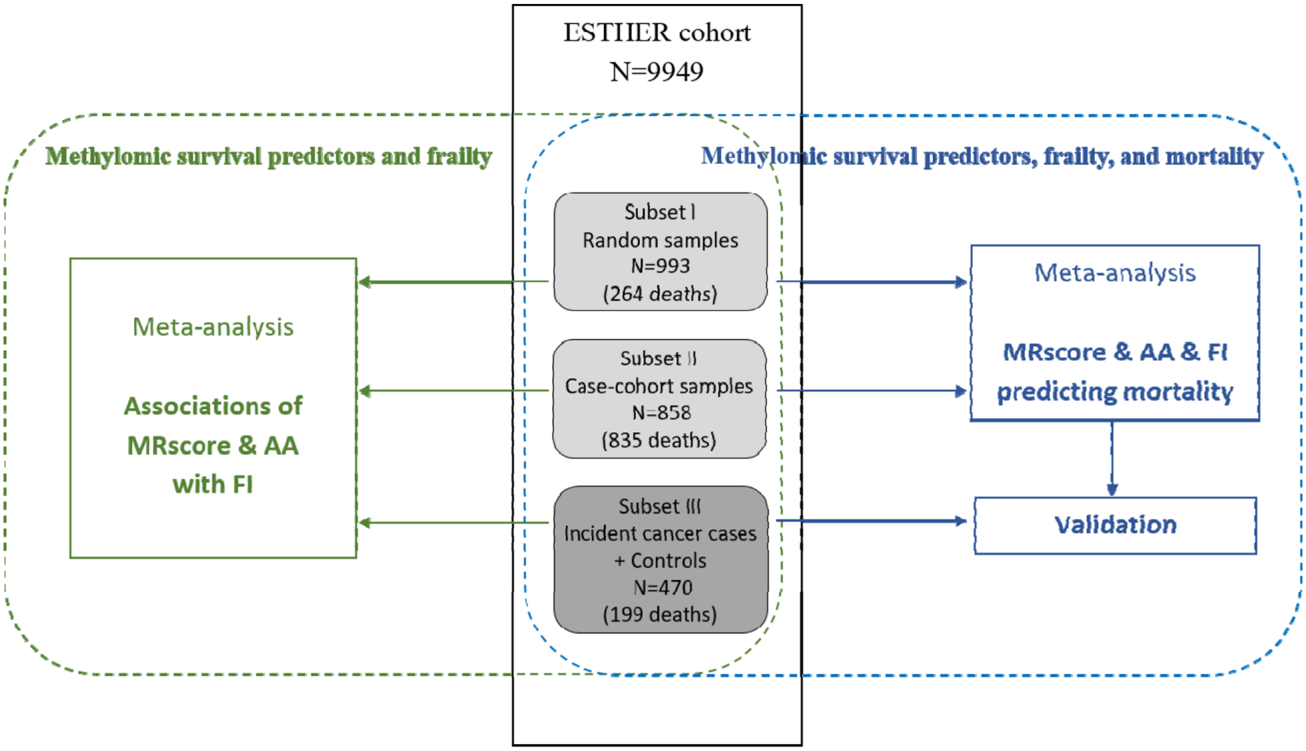

Figure S1. Study design and analysis flowchart.
